# Reference genes for accurate evaluation of expression levels in *Trichophyton interdigitale* grown under different carbon sources, pH levels and phosphate levels

**DOI:** 10.1038/s41598-019-42065-5

**Published:** 2019-04-03

**Authors:** Anita Ciesielska, Beata Oleksak, Paweł Stączek

**Affiliations:** Department of Microbial Genetics, Faculty of Biology and Environmental Protection, University of Łódź, Łódź, Poland

## Abstract

*Tinea pedis* is a type of dermatophytosis caused by anthropophilic keratinolytic fungi such as *Trichophyton interdigitale*. Quantitative reverse transcription PCR (RT-qPCR) is a reliable and reproducible technique for measuring changes in target gene expression across various biological conditions. A crucial aspect of accurate normalization is the choice of appropriate internal controls. To identify reference genes for accurate evaluation of expression levels in *T. interdigitale*, the transcription levels of eight candidate reference genes (*adp-rf, β-act*, *ef1-α*, *gapdh*, *psm1, sdha*, *rpl2* and *ubc)* and one target gene (*Tri m4*) were analysed by RT-qPCR after growing the dermatophyte under different environmental conditions. The results obtained from expression stability evaluations with NormFinder, geNorm, BestKeeper, and RefFinder software demonstrated that *adp-rf* and *psm1* were the most stable internal control genes across all experimental conditions. The present study constitutes the first report of the identification and validation of reference genes for RT-qPCR normalization for *T. interdigitale* grown under different environmental conditions resembling the conditions encountered by fungi during invasion of skin.

## Introduction

*Trichophyton interdigitale* is a keratinophilic and keratinolytic fungus belonging to the dermatophyte group^1^, and it is responsible for infections of the feet and toes (*tinea pedis*)^2^. Epidemiological studies have indicated that the incidence of dermatophytosis due to this fungus is rising and is not correlated with patient characteristics such as ethnicity or race^3^. However, *tinea pedis* is most frequently found in adults between 30 and 60 years old^4^, and this dermatophytosis is more common in men than in women^5^ and in developed countries^6^. Dermatophytes have been recorded worldwide with variations in epidemiology, distribution, incidence and target hosts from one location to another. Different conditions, such as geographic location, climate, health care quality, immigration status, hygiene culture, and socioeconomic status, may influence the development of dermatophyte infections^7^. Keratin is a structural protein of the *stratum corneum*, where dermatophytes typically infect, while elastin and collagen are the fibrous proteins of the extracellular matrix in the dermis that dermatophytes can penetrate during infection^8^. The availability of these proteins is necessary for the activation of signal transduction during dermatophyte infection. The degradation of keratin and other fibrous proteins releases high amounts of cysteine, proline, serine or lysine, and the metabolism of these amino acids leads to the secretion of ammonia, which raises the extracellular pH from acidic to alkaline values. Transcriptomic analyses suggest that during the first 48 h after infection^9^, the highest transcriptional activity of genes responsible for the synthesis of proteases and adhesins occurs, which allows colonization of the host tissue^10^. Analysis of changes in dermatophyte gene expression profiles under defined growth conditions can improve our knowledge of the mechanisms associated with the pathogenicity of dermatophytes and of the other biological properties of this group of pathogens. Information gathered during such study may be useful in the search for new therapeutic and prophylactic strategies. Quantitative reverse transcription PCR (RT-qPCR) is a powerful technique used to quantify the mRNA levels of different genes of interest under various experimental conditions. However, different experimental and technical variations can lead to incorrect data analysis. Therefore, it is necessary to establish a set of optimal reference genes before conducting target gene expression analysis. Due to the limited knowledge regarding reference genes useful for RT-qPCR analysis in dermatophytes^10^ and the particularly insufficient information on the complete genome sequence of *T. interdigitale*, eight reference genes, including *adp-rf* (ADP ribosylation factor), *β-act* (β-actin), *ef1-α* (elongation factor 1-alpha), *gapdh* (glyceraldehyde 3-phosphate dehydrogenase)*, psm1* (mitotic cohesion complex subunit Psm1)*, sdha* (succinate dehydrogenase complex flavoprotein subunit A), *rpl2* (ribosomal protein L2) and *ubc* (ubiquitin) (Table 1) were ultimately selected and evaluated in a *T. interdigitale* strain subjected to 13 different environmental conditions (Table 2). The selected candidate reference genes were chosen from among internal controls used in some species of fungi, including dermatophytes^10,11^, and in other eukaryotic organisms^9,10,12–15^. The expression stability of each candidate reference gene was calculated by the following algorithms: the geNorm module of qbase + (Biogazelle)^16^, NormFinder^17^, and BestKeeper^18^. The online comprehensive tool RefFinder was ultimately used to compare and rank the candidate reference genes.

**Table 1:** *Trichophyton interdigitale* candidate reference genes used for qRT-PCR.

*Gene symbol*/accession no.	Gene name	Primers (5′-3′) forward reverse	Length (bp)	Tm (°C)	C_t_ range	Efficiency (%)	R^2^
*adp-rf* (H101_06992)	ADP ribosylation factor	ATGCGAATTCTTATGGTCGG GTTGAATCCGATGGTGGG	105	60.5	18.12–21.89	100	0.9927
*β-act* (H101_06992)	β-actin	TGTTTTCCCATCCATTGTCG CATCACCAACATAGGAGTCC	117	60.5	15.20–19.80	104	0.99980
*ef1-α* (H101_03672)	elongation factor 1-alpha	GAGAAGTTCGAGAAGGAAGC GACGGTGACATTGTACTTGG	150	60.5	15.95–19.95	98	0.99924
*gapdh* (H101_04054)	glyceraldehyde 3-phosphate dehydrogenase	GAAGCCAGTCACCTACGA TGTATCCGAGAATACCCTTGA	80	60.5	16.56–22.64	107	0.99677
*psm1* (H101_01238)	mitotic cohesion complex 2	CGAGCTCTTCAATTTCAAGTC AAATGGGACGACTTGATTCC	150	60.5	18.80–22.45	101	0.99959
*sdha* (H101_02447)	succinate dehydrogenase complex flavoprotein subunit A	GAGGCTGGATTCAACACC TTGTGCATGTTTCCAAGAGC	104	60.5	16.01–19.99	108	0.99882
*rpl2* (H101_0787)	subunit Psm1 ribosomal protein L	GTGGATCTATCTTCACGGC ACAATCTTCTTCACGACACC	112	60.5	19.70–22.84	109	0.99901
*ubc* (H101_00343)	ubiquitin C	TGTCATGACTTGGAATGCTG TCCTCAAAATGCATCACGAG	87	60.5	22.78–26.32	103	0.99895

**Table 2:** *T. interdigitale* cultivation conditions in liquid minimal medium (MM) supplemented with different carbon sources, low-Pi MM, and low-Pi yeast extract medium (YEM).

Variant	Cultivation substrate	Cultivation pH	Cultivation conditions
MM-Cove	—	5.0	24 and 48 h, 28 °C, 200 rpm
MM-Cove	50 mM glucose	5.0	
MM-Cove	0.5% keratin	5.0	
MM-Cove	0.5%/1% keratin/soy protein	5.0	
MM-Cove	0.5% elastin	5.0	
MM-Cove	0.5% collagen	5.0	
MM-Cove	1% colloidal chitin	5.0	
Low-Pi MM	200 μM Pi	5.0	17 h, 37 °C, 200 rpm
Low-Pi MM	200 μM Pi	8.0	
Low-Pi MM	200 μM Pi	10.0	
Low-Pi YEM	700 μM Pi	5.0	
Low-Pi YEM	700 μM Pi	8.0	
Low-Pi YEM	700 μM Pi	10.0	

## Results

### Amplification efficiency and specificity of eight candidate reference genes

**Figure 1:**
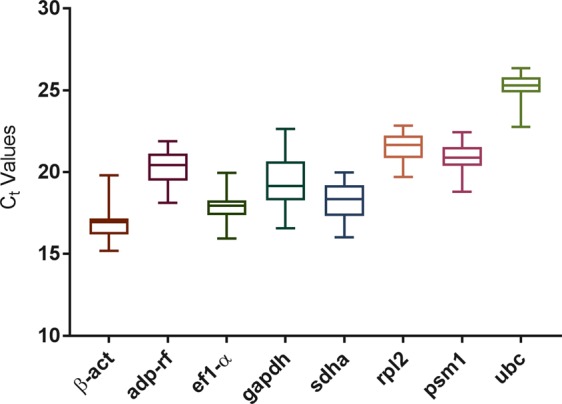
Expression levels of eight reference genes in *T. interdigitale*. The mean C_t_ values for all experimental conditions for each candidate reference gene are shown as box plot representations. Each box indicates the 25^th^ and 75^th^ percentiles. The line across the box represents the median, and the whisker caps show the maximum and minimum values.

The specificity of the primer sets was validated based on the identification of a single band of the expected size on 8% polyacrylamide gels and a single homogenous peak in melting curve analysis (Table 1, Supplementary Fig. S1A,B). The PCR efficiencies (E%) ranged from 99–110%, with correlation coefficient (R^2^) values varying from 0.996 to 0.999 (Table 1). The expression profiles of the eight reference gene candidates (Table 1) were analysed under control and experimental conditions by calculating the mean raw C_t_ value from three independent repetitions (Supplementary Table S1). As shown in Fig. 1, the C_t_ values of the eight candidate housekeeping genes ranged from 15.20 to 26.32 across all experimental conditions.

### Expression stability analyses

**Figure 2:**
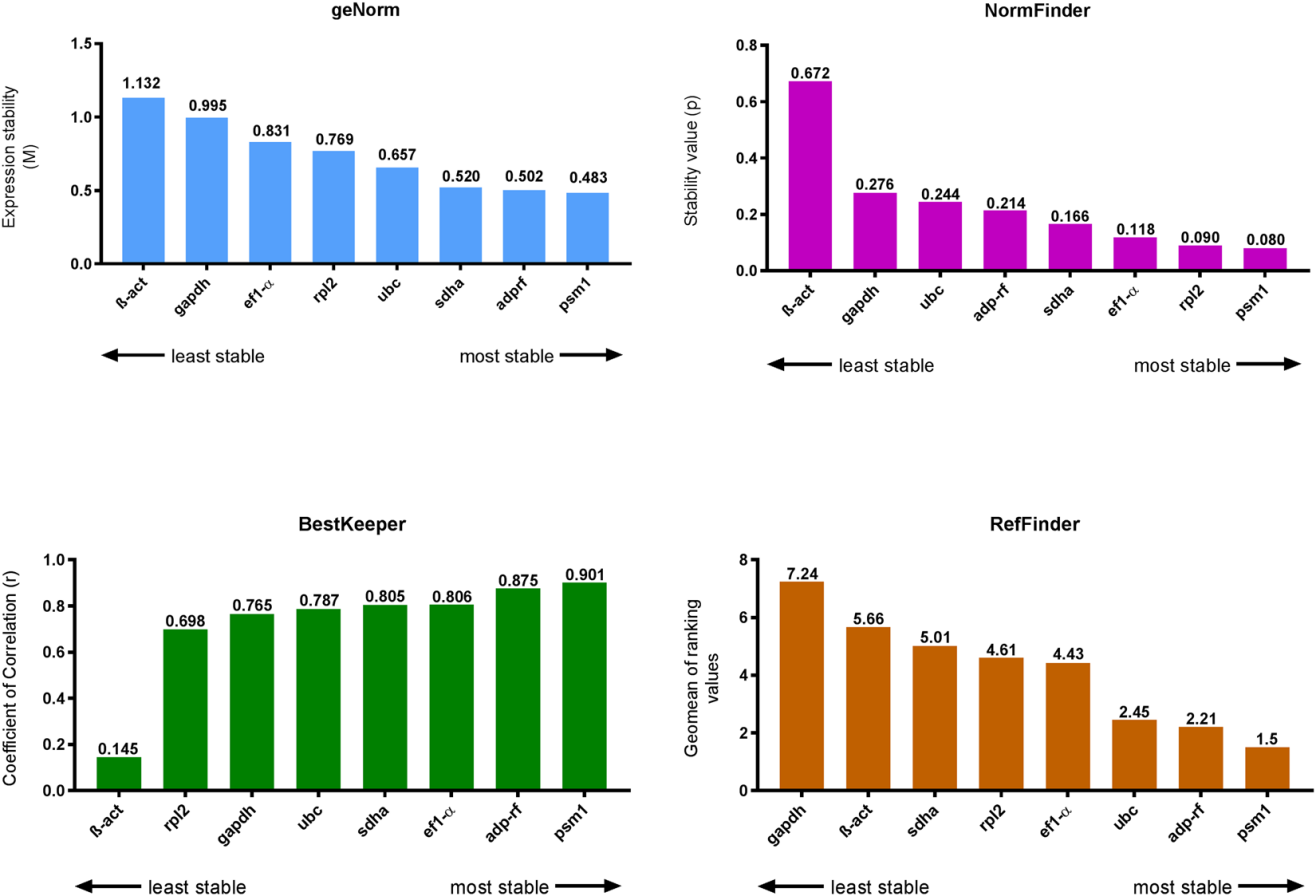
Ranking of *T. interdigitale* candidate reference genes by their expression stability, as determined by geNorm, NormFinder, BestKeeper, and RefFinder, for all experimental conditions.

**Figure 3:**
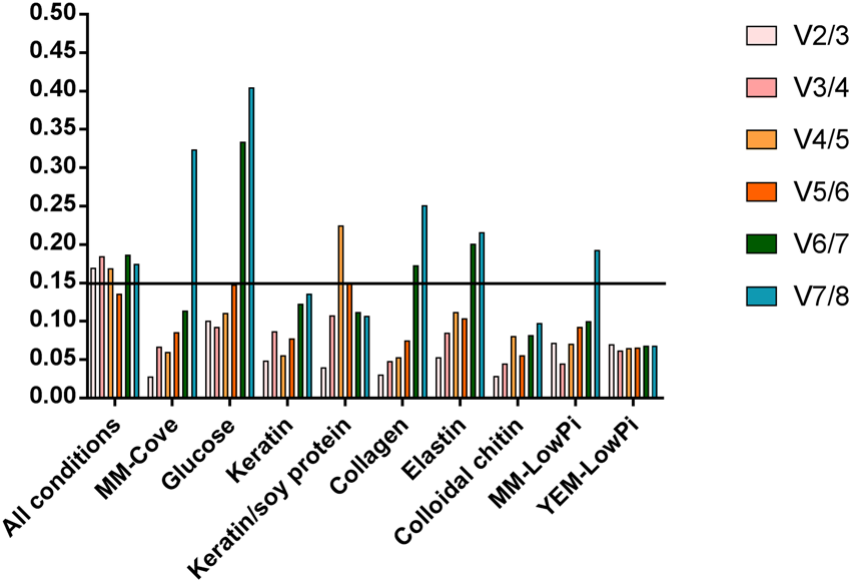
Pairwise variation (V_n_/V_n+1_) of the *T. interdigitale* candidate reference genes as determined by geNorm.

The GeNorm algorithm, which is a module of qbase + (Biogazelle), was used to evaluate the candidate reference genes based on their expression stability values (M-values) and pairwise variations (V_n_/V_n+1_). *Psm1* (M-value = 0.483), *adp-rf* (M-value = 0.502) and *sdha* (M-value = 0.520) were the most stable reference genes under all experimental conditions (Fig. 2). In contrast, the *β-act* gene had the highest M-value, with the lowest expression stability (M-value = 1.132) in all analysed samples (Fig. 2). The pairwise variation (V_n_/V_n+1_) results indicated that five reference genes (*psm1*, *adp-rf*, *sdha*, *ubc* and *rpl2*) should be used for reliable normalization (V_5/6_ = 0.136) (Fig. 3). The most stable reference genes (in order) among all chosen candidates for *T. interdigitale* under each experimental condition were as follows (Table 3): *ef1-α*, *rpl2*, *sdha*, *adp-rf*, *ubc*, *psm1*, *gapdh*, and *β-act* for control conditions (MM-Cove); *adp-rf, ubc, gapdh, psm1, ef1-α, sdha, rpl2*, and *β-act* for glucose supplementation; *ef1-α, ubc, sdha, psm1, rpl2, β-act, adp-rf*, and *gapdh* for keratin supplementation; *rpl2, gapdh, ef1 α-α, ubc, β-act, psm1, sdha*, and *adp-rf* for keratin and soy protein supplementation; *sdha, psm1, rpl2, adp-rf, ubc, β-act, ef1-α*, and *gapdh* for elastin supplementation; *ef1-α, psm1, adp-rf, rpl2, β-act, sdha, gapdh*, and *ubc* for collagen supplementation; *rpl2, psm1, adp-rf, β-act, ef1-α, ubc, gapdh*, and *sdha* for colloidal chitin supplementation; *adp-rf, ef1-α, β-act, sdha, psm1, ubc, rpl2*, and *gapdh* for low-Pi MM; and *β-act, adp-rf, psm1, ef1-α, gapdh, ubc, rpl2*, and *sdha* for low-Pi YEM. Furthermore, pairwise variation (V_n_/V_n+1_) calculation with a V-value < 0.15 showed that only two internal controls were sufficient for normalizing gene expression under all experimental conditions (Fig. 3).

**Table 3:** Gene expression stability (M-value) of each *Trichophyton interdigitale* candidate reference gene as analysed by geNorm for each experimental condition.

Rank	MM-Cove		MM-Cove + Glucose		MM-Cove + Keratin		MM-Cove + Keratin/soy protein		MM-Cove + Elastin		MM-Cove + Collagen		MM-Cove + Colloidal chitin		MM-Cove + Low-Pi MM		MM-Cove + Low-Pi YEM	
	gene	M	gene	M	gene	M	gene	M	gene	M	gene	M	gene	M	gene	M	gene	M
1	*ef1-α*	0.053	*adp-rf*	0.156	*ef1-α*	0.081	*rpl2*	0.060	*sdha*	0.103	*ef1-α*	0.053	*rpl2*	0.057	*adp-rf*	0.131	*β-act*	0.138
2	*rpl2*	0.064	*ubc*	0.159	*ubc*	0.088	*gapdh*	0.060	*psm1*	0.126	*psm1*	0.060	*psm1*	0.069	*ef1-α*	0.153	*adp-rf*	0.147
3	*sdha*	0.071	*gapdh*	0.207	*sdha*	0.108	*ef1-α*	0.080	*rpl2*	0.137	*adp-rf*	0.071	*adp-rf*	0.075	*β-act*	0.174	*psm1*	0.169
4	*adp-rf*	0.170	*psm1*	0.290	*psm1*	0.227	*ubc*	0.257	*adp-rf*	0.238	*rpl2*	0.132	*β-act*	0.128	*sdha*	0.190	*ef1-α*	0.216
5	*ubc*	0.222	*ef1-α*	0.396	*rpl2*	0.249	*β-act*	0.604	*ubc*	0.366	*β-act*	0.185	*ef1-α*	0.239	*psm1*	0.262	*gapdh*	0.270
6	*psm1*	0.320	*sdha*	0.559	*β-act*	0.322	*psm1*	0.705	*β-act*	0.452	*sdha*	0.274	*ubc*	0.272	*ubc*	0.364	*ubc*	0.318
7	*gapdh*	0.457	*rpl2*	1.067	*adp-rf*	0.476	*sdha*	0.727	*ef1-α*	0.724	*gapdh*	0.543	*gapdh*	0.358	*rpl2*	0.474	*rpl2*	0.374
8	*β-act*	0.992	*β-act*	2.01	*gapdh*	0.628	*adp-rf*	0.759	*gapdh*	0.975	*ubc*	0.909	*sdha*	0.464	*gapdh*	0.745	*sdha*	0.423

**Figure 4:**
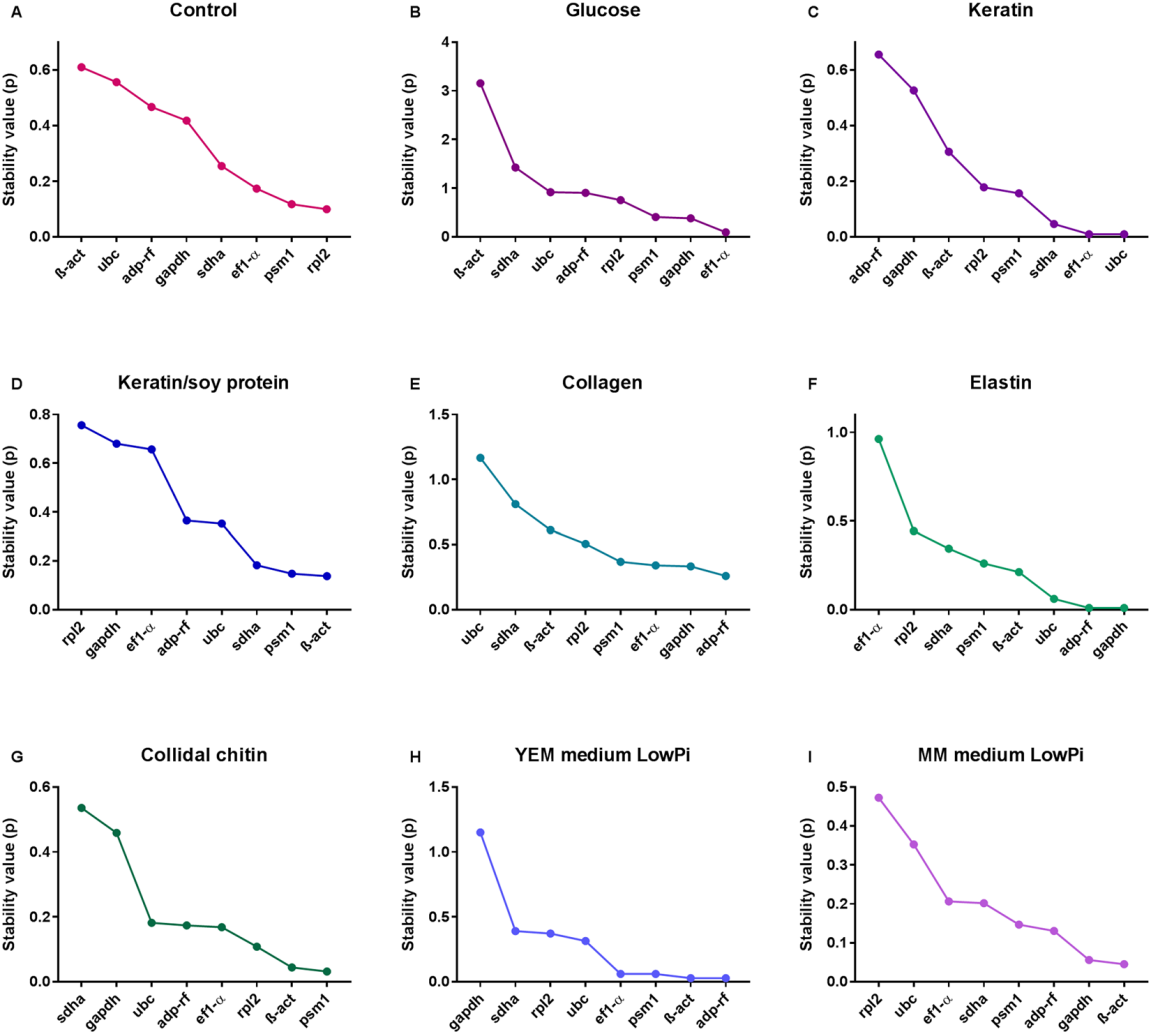
Ranking of *T. interdigitale* candidate reference genes as determined by NormFinder for all experimental conditions.

According to NormFinder^17^, across all experimental conditions, *psm1* had the lowest stability value (SV = 0.080) (Fig. 2). *Psm1* and *rpl2* constituted the best combination of internal control genes with SV = 0.061 under all experimental conditions. *Psm1* was found to be the most stably expressed gene in the presence of colloidal chitin (SV = 0.032) (Fig. 4G), and *rpl2* was the most stable gene under control conditions (SV = 0.100) (Fig. 4A). β-*act* was the most stable gene in the medium supplemented with keratin and soy protein (SV = 0.138) (Fig. 4D) and under low-Pi conditions in MM (SV = 0.045) (Fig. 4I). In the case of collagen supplementation and under the low-Pi condition in YEM, NormFinder calculations revealed that *adp-rf* had the lowest stability values, with SV = 0.259 (Fig. 4E) and SV = 0.028 (Fig. 4H), respectively. *Ef1-α*, *ubc* and *gapdh* were the most stably expressed genes in the presence of glucose (SV = 0.096) (Fig. 4B), keratin (SV = 0.010) (Fig. 4C), and elastin (SV = 0.010) (Fig. 4F).

Assessment of the expression variation of the candidate reference genes using the BestKeeper algorithm^18^ revealed that seven of the genes had standard deviation values defined as acceptable [0.5 < SD[±C_t_] ≤ 1.00], while *gapdh* had an unacceptable standard deviation, as it was higher than 1.0 (SD = 1.32) (Table 3). Analyses showed also significant expression correlations with the BestKeeper index, which is the geometric mean of the C_t_ values of the analysed genes (correlation coefficient r = 0.698–0.901), except for the *β-act* gene (r = 0.145). The expression of all genes correlated with the BI with p values < 0.001, except the *β-act* gene (p-value = 0.543). The overall order of the most stable genes based on BestKeeper was *psm1*, *adp-rf*, *ef1-α*, *sdha*, *ubc* and *rpl2* (Fig. 2, Table 4).

**Table 4:** Descriptive statistics for the candidate reference genes calculated using BestKeeper.

N	*β-act*	*adp-rf*	*ef1-a*	*gapdh*	*sdha*	*rpl2*	*psm1*	*ubc*
	60	60	60	60	60	60	60	60
GM (C_t_)	16.80	20.32	17.78	19.30	18.18	21.51	20.74	25.11
AM (C_t_)	16.82	20.34	17.81	19.37	18.22	21.53	20.76	25.12
Min (C_t_)	15.2	18.12	15.95	16.56	16.01	19.7	18.8	22.78
Max (C_t_)	19.8	21.89	19.95	22.64	19.99	22.84	22.45	26.36
SD ( ± C_t_)	0.69	0.84	0.74	**1.32**	0.89	0.71	0.79	0.69
CV (% C_t_)	4.15	4.14	4.16	6.82	4.91	3.31	3.82	2.77
Min (fold change)	−3.03	−4.59	−3.57	−6.72	−4.51	−3.53	−3.84	−5.03
Max (fold change)	7.99	2.96	4.47	10.05	3.49	2.49	3.26	2.37
SD ( ± fold change)	1.62	1.79	1.67	2.50	1.85	1.64	1.73	1.62
BI Index (r)	0.145	**0.875**	**0.806**	0.765	**0.805**	**0.698**	**0.901**	**0.787**
p-value	0.543	0.001	0.001	0.001	0.001	0.001	0.001	0.001

n, number of samples (three biological replicates and 30 different conditions); GM (C_t_), geometric mean of C_t_; AM (C_t_), arithmetic mean of C_t_; Min (C_t_) and Max (C_t_), extreme values of C_t_; SD (±C_t_), standard deviation of C_t_; CV (% C_t_), coefficient of variation expressed as a percentage of the C_t_ value; Min (fold change) and Max (fold change), extreme value of the expression level expressed as the absolute fold change of down- or upregulation; SD (±fold change), standard deviation of the absolute fold change; BI Index (r), correlation between BestKeeper index and the contributing gene.

In the final step, RefFinder, a free online tool for the identification of stable reference genes that integrates all methods applied in the present study, was used to generate a final ranking of the eight reference genes according to their geomean ranking values. As shown in Fig. 2, *psm1* and *adp-rf* were ranked as the best reference genes for measuring target gene expression levels under the chosen conditions.

### Stability and validation of *adp-rf* and *psm1* as reference genes

**Figure 5:**
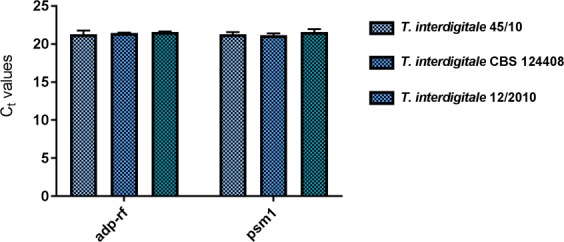
Stability of *adp-rf* and *psm1* reference gene expression in three *T. interdigitale* strains cultivated in control medium (MM-Cove) and MM-Cove supplemented with keratin. The gene expression levels are presented as the average C_t_ values. The *adp-rf* (*p* = 0.93, ANOVA) and *psm1* (*p* = 0.89, ANOVA) gene expression levels were not significantly different across the analysed culture conditions. The error bars indicate the standard error.

**Figure 6:**
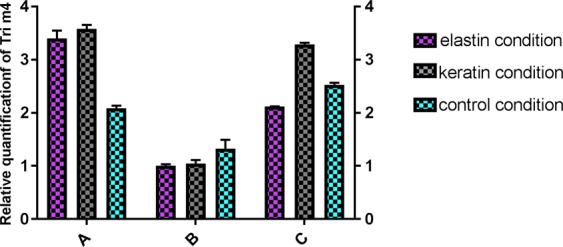
Relative quantification of *Tri m4* gene expression in control, keratin and elastin protein samples using different reference gene variants: (**A**) (*p* = 0.55, ANOVA), which included the two most stable reference genes, *adp-rf* and *psm1*; (**B**) (*p* = 0.46, ANOVA), which included the two least stable reference genes, *β-act* and *gapdh*; and (**C**) (*p* = 0.36, ANOVA), which included all candidate reference genes. The error bars indicate the standard error.

To confirm *adp-rf* and *psm1* as the most stable reference genes, their expression was compared in the *T. interdigitale* CBS 124408 reference strain and two clinical isolates: *T. interdigitale* 12/2010 and *T. interdigitale* 45/10. These three strains of *T. interdigitale* were incubated at 28 °C for 48 h in control medium and in medium supplemented with keratin. The obtained C_t_ values (Fig. 5) for the *adp-rf* and *psm1* genes were not significantly different under both conditions (*p*_psm1_ = 0.93; *p*_adp-rf_ = 0.89, ANOVA) (Fig. 5), which confirmed that these reference genes can be used for accurate expression level evaluation in various *T. interdigitale* strains. To confirm the reliability of *adp-rf* and *psm1* as reference genes for RT-qPCR normalization, the expression of *Tri m4* was examined^19^. *Tri m4* is known as an aminopeptidase gene whose expression increases in the presence of keratin and elastin, which suggests that the product of this gene may play an important role as a virulence factor^19^. The validation was performed using templates from the *T. interdigitale* 45/10 strain incubated at 28 °C for 48 h in control medium (MM-Cove) and in medium supplemented with keratin or elastin. Three different sets of reference genes were analysed: set A included the most stable reference genes (*adp-rf* and *psm1*), set B included the least stable reference genes (*β-act* and *gapdh*), and set C included all eight candidate reference genes. The relative expression of the target gene was determined using the 2^−ΔΔCt^ method^20^. As shown in Fig. 6, an increase in *Tri m4* transcript levels in *T. interdigitale* growing in the presence of keratin or elastin in relation to control conditions was noticed only when the *adp-rf* and *psm1* reference genes (set A) previously selected by all four algorithms were used.

## Discussion

Quantitative reverse transcription PCR (RT-qPCR) is an efficient method for analysing target gene expression but requires comprehensive normalization with properly selected reference genes. The internal controls should have relatively stable expression levels in different types of cells or tissues, and their expression should be constant under various growth conditions^21^. Many reports on target gene expression analysis have used a single housekeeping gene for RT-qPCR normalization, such as *18S rRNA*, *gapdh*, *β-act, β-tub* or *ef1-α*^22^; these genes regulate basic cellular functions. Such an approach has a strong tradition and history of use since the introduction of reverse-transcription-based assays. However, since 2009, according to the MIQE Guidelines: Minimum Information for Publication of Quantitative Real-Time PCR Experiments^21^, no single reference gene should be used to quantify target gene expression under different conditions, and candidates for reference genes should be carefully selected for each study to comply with these guidelines. Numerous studies have shown that the expression of the above housekeeping genes can vary between individual tissues or experimental conditions such that no universal reference gene can be used in all situations^18,23–27^. Moreover, a reference gene with stable expression in one organism may not be suitable for normalization of gene expression in another organism, even a closely related organism. Additionally, genes such as *18S rRNA* and *28S rRNA*, despite their stability, are often expressed at very high levels and thus should not be used as internal controls^28,29^. To avoid biased normalization, following the MIQE guidelines^21^, the use of multiple candidate reference genes is highly recommended to obtain reliable RT-qPCR results. Based on the limited literature reports regarding the analysis of gene expression in dermatophytes^10,11^, supplemented with information on reference genes used in studies on other eukaryotes^9,12,13^, 12 genes, including *adp-rf* (ADP ribosylation factor), *β-act* (β-actin), *β-tub* (β-tubulin)*, ef1-α* (elongation factor 1-alpha), *gapdh* (glyceraldehyde 3-phosphate dehydrogenase)*, mbp1* (multiubiquitin chain-binding protein 1), *fis1* (mitochondrial fission 1 protein), *psm1* (mitotic cohesion complex subunit Psm1)*, rGTPa* (rho GTPase activating-protein 5), *rpl2* (ribosomal protein L2), *sdha* (succinate dehydrogenase complex flavoprotein subunit A) and *ubc* (ubiquitin) (Table 1 and Supplementary Table S2), were selected as putative candidates based on a BLAST search of available *T. interdigitale* genomic sequences. Despite several attempts at primer modification, only 8 candidate amplification products were obtained, and these genes were used in further studies. Unfortunately, only three genomes of this species are currently available in the databases, of which two are at a scaffold level, while the third is at a contig level. An in-depth analysis of these genomes revealed the presence of regions of predicted sequences, indicating that the full nucleotide sequences have yet to be established (https://www.ncbi.nlm.nih.gov/genome/genomes/44693). It can therefore be assumed that both these problems and others related to the difficulties in correctly determining the taxonomic affiliation of many strains of *T. interdigitale*, as described in the literature^30^ (which may affect the correct genome assembly of this species), were responsible for the unsuccessful attempts to develop a larger number of correct primers, making it impossible to test a greater number of putative reference genes.

In this study, the geNorm, NormFinder, BestKeeper and RefFinder algorithms were used to evaluate the selected candidate reference genes as internal controls for analysis of target gene expression in *T. interdigitale* growing under different environmental stimuli, such as supplementation with various carbon sources, low Pi, and different pH values^10^. Some of these circumstances have been suggested to promote adhesion to the host tissue and are essential for the expression of specific genes associated with adaptation and interactions between *T. interdigitale* and its host. Our study is the first report on the identification and validation of reference genes for *T. interdigitale* that indicates *psm1* and *adp-rf* as the most stable genes among the analysed candidates (Fig. 2).

Mitotic cohesion complex ATPase subunit (*psm1*) is involved in mitotic cohesion loading/unloading and is required for the cohesion of sister chromatids after DNA replication. In addition, ADP-ribosylation factor (*adp-rf*) is a ubiquitous GTP-binding protein essential for mitotic growth. These two candidates were found in the present study to be reliable internal controls for accurate expression level analysis of target genes of *T. interdigitale* growing under adhesion-inducing conditions.

Llanos *et al*.^31^ showed that according to geNorm analysis, *psm1*, *ubcB* (ubiquitin carrier protein) and *sac7* (Rho GTPase activator) were ranked as the most stably expressed reference genes in the fungus *Talaromyces versatilis* grown under various conditions, such as in the presence of different carbon sources; under different temperatures and pH levels; and under salt stress and carbon/nitrogen starvation. However, in our previous report on the validation of reference genes for the dermatophyte *Microsporum canis*^11^, *psm1* was classified in the group of unstable reference genes. To date, there have been only two studies on the validation of *psm1* as a stably expressed reference gene in RT-qPCR analysis.

The ADP ribosylation factor gene (*adp-rf*) was found to be the best reference gene for analyses of target transcript levels in an exotic invasive insect, *Leptinotarsa decemlineata*^32^; the melon *Cucumis melo* L.^33^; the cereal wheats *Triticum* spp.^12^, the Pacific oyster, *Crassostrea gigas*^34^; the monkey *Macaca fascicularis*^35^; and the desert willow shrub, *Salix psammophila*^36^. Again, our previous study^11^ demonstrated that the expression stability of *adp-rf* was low in *M. canis*. Furthermore, the *β-act* gene, which encodes a cytoskeletal protein involved in many cellular processes, and the *gapdh* gene, which encodes an enzyme of the glycolytic pathway, are often used as reliable reference genes in expression analyses^37^; however, these genes were in the group of the least stable reference genes in the present study. On the other hand, in the search for reliable reference genes for RT-qPCR analysis of target gene expression in *M. canis*, the *β-act* gene was classified as one of the three most stable genes^11^. The present and the previous results^11^ of our team confirmed that the stability of housekeeping gene expression should be verified for each condition and each particular species (Table 3, Fig. 4), which again highlights the fact that there is no ideal and universal internal control gene for RT-qPCR analysis.

To validate the reference genes selected by the four algorithms, the genes were used as reference genes for the measurement of the relative expression of *Tri m4*, a gene that encodes aminopeptidase and is known to be upregulated in the presence of keratin and elastin as inducers^19^. Elevated *Tri m4* expression was detected in *T. interdigitale* growing under inducing conditions only when the two internal controls *psm1* and *adp-rf*, which were selected as the most stable internal controls by the four algorithms, were used in combination (Fig. 6). Upregulation was detected neither for the least stable pair of reference genes (set B) nor for the whole set of eight candidate genes (set C). These results clearly confirmed that the chosen best pair of internal control genes can be preferentially used for RT-qPCR normalization in the case of *T. interdigitale* grown under the described experimental conditions.

## Conclusion

The present study was the first attempt to identify and validate *T. interdigitale* internal control genes. The *psm1* and *adp-rf* genes were found to be the most stable reference genes appropriate for gene expression analysis in *T. interdigitale*. The use of these genes as internal controls may further improve the robustness of RT-qPCR for *T. interdigitale* grown under adhesion-inducing conditions.

## Materials and Methods

### Reference gene selection and primer design

Twelve putative candidate reference genes (*adp-rf*, *β-act*, *β-tub, ef1-α*, *fis1*, *gapdh*, *mbp1*, *psm1*, *sdha*, *rpl2*, *rGTPa* and *ubc*) (Table 1 and Supplementary Table S2) were chosen in the present study based on the NCBI database (http://www.ncbi.nlm.nih.gov) and our previous study^11^. Primers were designed and theoretically evaluated using Primer 3 software^38^. PCR products within the 80–150 bp range were obtained only in the case of 8 candidates (*adp-rf*, *β-act*, *ef1-α*, *gapdh*, *psm1*, *sdha*, *rpl2* and *ubc*), and these genes were analysed by PCR in a Gradient Thermal Cycler T1000 (BioRad) (Table 1, Supplementary Fig. S1B).

### Fungal material and growth conditions

The *Trichophyton interdigitale* 45/10 strain, isolated from *tinea pedis* of a 42-year-old man, was used in all RT-qPCR analyses. *Trichophyton interdigitale* CBS 124408 (a reference strain from the CBS-KNAW Collection, Utrecht, The Netherlands) and *Trichophyton interdigitale* 12/2010, a clinical isolate from the *onychomycosis* case of a 61-year-old man, were used in the evaluation step of the reference genes. The clinical strains were chosen from the collection maintained in the Department of Microbial Genetics, Faculty of Biology and Environmental Protection, University of Łódź, Poland. PCR-RFLP analysis of the ITS1-5.8S-ITS2 region followed by sequencing was performed for standard mycological identification^39^. Germinated conidia of the *T. interdigitale* strain (approximately 10^7^ cells/ml)^40^ were incubated separately in minimal liquid medium (MM-Cove)^41^ under 7 different conditions (Table 2), in low-Pi MM, and in YEM (yeast extract medium) under 3 different conditions^42^ (Table 2).

### RNA extraction, cDNA synthesis and quantitative reverse transcription PCR

Total RNA was extracted using an RNeasy Plant Mini Kit (Qiagen) following the manufacturer’s protocol. RNA integrity was verified by electrophoretic and spectrophotometric (NanoPhotometerPearl Version 1.0, IMPLEN) analyses, according to the MIQE guidelines^21^ for RT-qPCR. RNA samples with A260/A280 ratios between 1.9 and 2.1 were used for further analysis. First-strand cDNA was synthesized using 2 μg of total RNA (DNA-free), RevertAid Transcriptase (Thermo Scientific) and random hexamer primers (Thermo Scientific) following the manufacturer’s protocol. The qRT-PCR reactions were conducted on a RotorGene Q System (Qiagen) based on a method described previously^11^ using SsoAdvanced Universal SYBR® Green Supermix (2X) (Bio-Rad). The reactions were subjected to an initial step of 95 °C for 1 min followed by 40 cycles at 95 °C for 20 s, 60.5 °C for 20 s, at 72 °C for 15 s. Melting curve analysis was performed by heating the amplicon from 72 °C to 95 °C.

### Data analysis

The expression stability of the candidate reference genes in *T. interdigitale* was analysed using four bioinformatic tools: geNorm^16^, NormFinder^17^, BestKeeper^18^ and RefFinder (http://leonxie.esy.es/RefFinder/). The geNorm tool was used to calculate the gene expression stability according to the M-value, which is defined as the average pairwise variation with all other tested candidate reference genes. The algorithm recommends selecting genes with M-values below 1.0 to ensure the choice of the most stably expressed internal control genes^11^. Moreover, Vandesompele *et al*.^16^ suggested that an M-value lower than 0.5 indicates very good stability of expression. GeNorm also suggests that the best combination of reference genes based on pairwise variations (V_n_/V_n+1_) between two sequential normalization factors (NF_n_ and NF_n+1_) has a V-value < 0.15. NormFinder is a VBA tool for Microsoft Excel used to calculate stability values (SVs) by combining intra- and inter-group variations in reference gene expression^17^. Lower SV values correspond to lower variations and, hence, higher stability of the reference genes. BestKeeper ranks the candidate reference genes according to their correlation coefficients (r values) for correlation with the BestKeeper Index (BI), which is the geometric mean of the C_t_ values of the candidate reference genes determined by calculating the standard deviation (SD) and coefficient of variance (CV)^18^. The online tool RefFinder was used to measure the geometric mean of the attributed weights for the overall final ranking. Box-and-whisker plots were drawn and one-way ANOVA was performed using GraphPad Prism version 7.00 for Windows (GraphPad Software, La Jolla, California, USA).

## Supplementary information

Supplementary Informations
