# Supplementary material for: Reference genes for accurate evaluation of expression levels in Trichophyton interdigitale grown under different carbon sources, pH levels and phosphate levels
Source: Sci Rep. 2019 Apr 3;9:5566. doi: 10.1038/s41598-019-42065-5 (PMC6447595; doi:10.1038/s41598-019-42065-5)

SUPPLEMENTARY INFORMATIONS

Reference genes for accurate evaluation of expression levels in *Trichophyton interdigitale* grown under different carbon sources, pH levels and phosphate levels

Anita Ciesielska*, Beata Oleksak and Paweł Stączek

Department of Microbial Genetics, Faculty of Biology and Environmental Protection, University of Łódź, Banacha 12/16, 90-237 Łódź, Poland

*Corresponding author: anita.ciesielska@biol.uni.lodz.pl

TABLES

Supplementary Table S1. Averaged raw C_t values of the *T. interdigitale* samples in different experimental conditions

Supplementary Table S2. *Trichophyton interdigitale* reference genes selected as putative candidates for RT-qPCR but not used in this study

FIGURE

Supplementary Figure S1. Melting curves of the eight *T. interdigitale* candidate reference genes show single peaks (A). 8% polyacrylamide gel electrophoresis indicated the amplification of a single product of the expected size for nine reference genes (B)

Table S1 Averaged raw C_t values of the *T. interdigitale* samples in different experimental conditions.

Experimental conditions	*β-act*	*adp-rf*	*ef1-α*	*gapdh*	*sdha*	*rpl2*	*psm1*	*ubc*
**C-24h,28°C**	16.87	18.43	16.2	16.56	16.31	19.7	18.91	23.24
**C-48h,28°C**	15.49	20.88	18.26	19.82	18.46	21.7	20.43	25.83
**G-24h,28°C**	19.8	18.12	15.95	17.26	16.01	20.51	18.8	22.78
**G-48h,28°C**	15.3	21.15	17.93	19.87	19.87	20.02	21.17	25.83
**Ker-24h,28°C**	15.8	18.73	16.45	16.85	16.78	20.14	19.51	24.26
**Ker-48h,28°C**	17.19	21.89	18.25	20.5	18.77	22.51	21.85	26.02
**KS-24h,28°C**	17.41	20.8	17.95	18.93	19.61	22.26	21.83	25.35
**KS-48h,28°C**	16.19	19.27	18.36	19.51	18.34	22.84	20.6	25.26
**Col-24h,28°C**	16.78	20.36	17.56	18.47	18.36	22.19	20.36	23.68
**Col-48h,28°C**	15.2	19.31	16.4	18.8	16.44	20.78	19.16	25.38
**E-24h,28°C**	16.22	20.28	17.91	17.94	18.22	21.99	20.71	25.17
**E-48h,28°C**	17.01	20.39	19.95	20.72	17.86	21.47	20.48	25.71
**CH-24h,28°C**	17.06	20.24	18.83	22.64	17.13	21.61	20.93	25.22
**CH-48h,28°C**	17.14	19.98	19.29	21.91	18.2	21.44	20.83	25.7
**5Y-17h, 37°C**	17.04	21.22	17.5	19.09	18.58	22.17	21.59	25.23
**8Y-17h,37°C**	17.87	21.89	18.06	19.19	19.99	22.57	22.45	26.32
**10Y-17h,37°C**	16.89	20.96	17.7	18.64	19.35	21.1	21.79	24.84
**5M-17h,37°C**	16.66	20.5	17.34	18.19	18.1	22.3	21.13	25.39
**8M-17h,37°C**	17.04	21.15	18.15	21.19	18.71	21.56	21.36	25.01
**10M-17h,37°C**	17.63	21.42	18.27	21.46	19.35	21.9	21.44	26.36

C-MM-Cove medium (control medium); **G**-MM-Cove+glucose; **Ker**-MM-Cove+keratin; **KS**-MM-Cove+keratin/soy protein; **Col**-MM-Cove+collagen; **E**- MM-Cove+elastin; **CH**-MM-Cove+colloidal chitin; **5Y**-YEM Low Pi medium pH 5.0; **8Y**- YEM Low Pi medium pH 8.0; **10Y**- YEM Low Pi medium pH 10.0; **5M**-MM-Cove LowPi medium pH 5.0; **8M**- MM-Cove LowPi medium pH 8.0; **10M**- MM-Cove LowPi medium pH 10.0.

Table S2 *Trichophyton interdigitale* reference genes selected as putative candidates for RT-qPCR but not used in this study.

Gene symbol/ **accession no.**	**Gene name**	**Primers (5'-3') forward reverse**	**Tm (°C)**
β-tub **(H101_01775)**	β-tubulin	CCATCTTCCGTGGTAAGGT<br>TCTGGACATTGTTGGGAATC<br>GTATGATGGCCACTTTCTCC<br>CAGATGTTATACAAAGCCTCG	60.5
mbp1 **(H101_03768)**	multiubiquitin chain binding protein	GACTACCTCCCCACAAGA<br>CTGACTGTGGGTGTGCTT<br>CCCTGACTGAAGATATTGGC<br>TGTTTAAGTGCTAGGTAGGC<br>ACTACCTCCCCACAAGATTC<br>CAATATCTTCAGTCAGGGTAAC	60.5
fis1 **(H101_04054)**	mitochondria fission 1 protein	AAGTTCAACTATGCCTGGGG<br>GAAGATCTCAGACAGCAGC<br>GTACGAGAAAGAAGGCGAAT<br>GGCCGGGAGTTTGATTTTAT	60.5
rGTP **(H101_06687)**	rho GTPase activator	CTTGAAGGAGAAAGCCACC<br>GGTCTGGGGAGTTGAATATC<br>TACAACTACACCCTCTCCAG<br>GCTAGCACTGCGTCCTTC	60.5

Figure S1 Melting curves of the eight *T. interdigitale* candidate reference genes show single peaks (A). 8% polyacrylamide gel electrophoresis indicated the amplification of a single product of the expected size for nine reference genes (B).

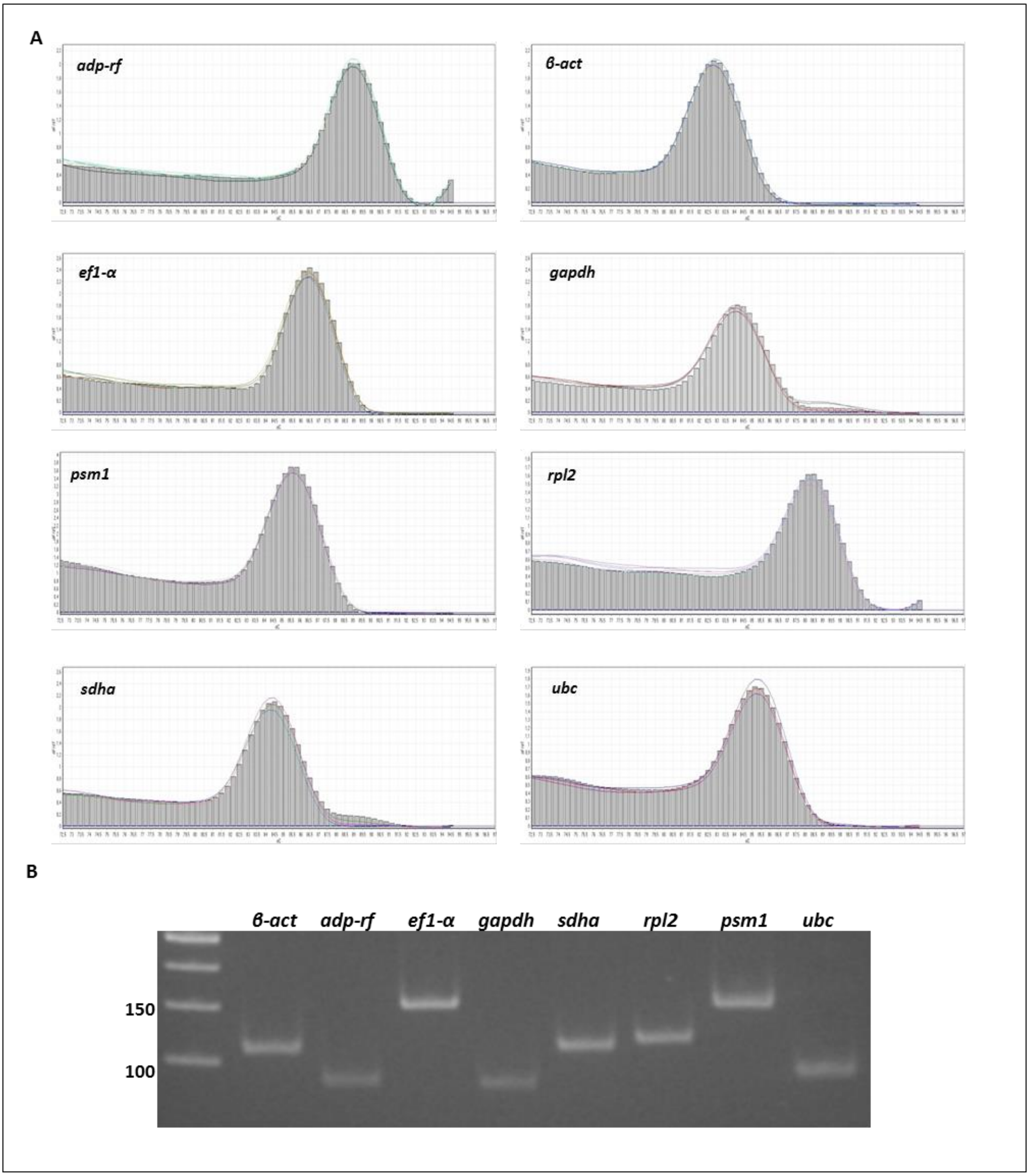

Supplement: Supplementary file 1 — Supplementary Informations [file 41598_2019_42065_MOESM1_ESM.pdf]
